# Prevalence of bat viruses associated with land-use change in the Atlantic Forest, Brazil

**DOI:** 10.3389/fcimb.2022.921950

**Published:** 2022-12-09

**Authors:** Elizabeth H. Loh, Alessandra Nava, Kris A. Murray, Kevin J. Olival, Moisés Guimarães, Juliana Shimabukuro, Carlos Zambrana-Torrelio, Fernanda R. Fonseca, Daniele Bruna Leal de Oliveira, Angélica Cristine de Almeida Campos, Edison L. Durigon, Fernando Ferreira, Matthew J. Struebig, Peter Daszak

**Affiliations:** ^1^ Division of Natural Sciences and Mathematics, Transylvania University, Lexington, KY, United States; ^2^ Durrell Institute of Conservation and Ecology, School of Anthropology and Conservation, University of Kent, Canterbury, United Kingdom; ^3^ Instituto Leônidas e Maria Deane – Fiocruz Amazônia, Manaus, Amazonas, Brazil; ^4^ MRC Unit The Gambia at London School of Hygiene and Tropical Medicine, Fajara, Gambia; ^5^ EcoHealth Alliance, New York, NY, United States; ^6^ Departamento de Recursos Naturais, Faculdade de Ciências Agronomicas, Universidade Estadual Paulista, Botucatu, Brazil; ^7^ Departamento de Medicina Veterinária Preventiva e Saúde Animal da Faculdade de Medicina Veterinária e Zootecnia da Universidade de São Paulo, São Paulo, Brazil; ^8^ Department of Environmental Science and Policy, George Mason University, Fairfax VA, United States; ^9^ Departamento de Microbiologia, Instituto de Ciências Biomédicas-II, Universidade de São Paulo, São Paulo, Brazil

**Keywords:** viral richness, diversity, bat host, deforestation, land-use change, viral prevalence

## Abstract

**Introduction:**

Bats are critical to maintaining healthy ecosystems and many species are threatened primarily due to global habitat loss. Bats are also important hosts of a range of viruses, several of which have had significant impacts on global public health. The emergence of these viruses has been associated with land-use change and decreased host species richness. Yet, few studies have assessed how bat communities and the viruses they host alter with land-use change, particularly in highly biodiverse sites.

**Methods:**

In this study, we investigate the effects of deforestation on bat host species richness and diversity, and viral prevalence and richness across five forested sites and three nearby deforested sites in the interior Atlantic Forest of southern Brazil. Nested-PCR and qPCR were used to amplify and detect viral genetic sequence from six viral families (corona-, adeno-, herpes-, hanta-, paramyxo-, and astro-viridae) in 944 blood, saliva and rectal samples collected from 335 bats.

**Results:**

We found that deforested sites had a less diverse bat community than forested sites, but higher viral prevalence and richness after controlling for confounding factors. Viral detection was more likely in juvenile males located in deforested sites. Interestingly, we also found a significant effect of host bat species on viral prevalence indicating that viral taxa were detected more frequently in some species than others. In particular, viruses from the *Coronaviridae* family were detected more frequently in generalist species compared to specialist species.

**Discussion:**

Our findings suggest that deforestation may drive changes in the ecosystem which reduce bat host diversity while increasing the abundance of generalist species which host a wider range of viruses.

## Introduction

Emerging viruses with wildlife origins are a significant threat to global health (e.g. Ebolaviruses, SARS and MERS coronaviruses) (Jones et al., 2008). Analyses of recent emerging infectious disease (EID) events show that anthropogenic changes including land-use change (e.g. habitat degradation, deforestation, forest fragmentation), intensification of food production, and global trade and travel are key factors in disease emergence (Loh et al., 2015; Allen et al., 2017; Rulli et al., 2017; Reaser et al., 2022). Further, nearly one-third of all EIDs, and a higher proportion of zoonoses, are associated with land-use change specifically (Loh et al., 2015). This suggests that increasing and/or novel interactions among hosts, vectors and pathogens following land-use change are significant contributors to disease emergence.

In tropical and subtropical environments, the pace of land-use change is unprecedented and continues to increase globally as demand for natural resources grows (Song et al., 2018). Bats are globally threatened, with 15% of bat species being listed as threatened or vulnerable, and habitat loss in the tropics is a major driver of population declines (Frick et al., 2020). Land-use change has also been associated with the emergence of many recent zoonotic diseases (Gibb et al., 2020). Yet, the relationship between land-use change and disease emergence is poorly understood. Recent studies have hypothesized that land-use change may increase the risk of disease emergence through more frequent human-animal interactions, or by influencing pathogen diversity, either directly by changing pathogen prevalence and/or diversity, or indirectly *via* impacts on host assemblages (Bradley et al., 2008; Vittor et al., 2009; Murray and Daszak, 2013; Rulli et al., 2017). However, mechanistic studies have tended to focus on how abundance and prevalence of specific pathogens, or their vectors and hosts, vary over the landscape (Ostfeld and Keesing, 2000; LoGiudice et al., 2003; Kilpatrick et al., 2006a; Kilpatrick et al., 2006b; Bradley et al., 2008; Vittor et al., 2009). Others have used meta-analyses to try to identify generality and mechanisms involved (Salkeld et al., 2013; Gottdenker et al., 2014; Civitello et al., 2015). Few empirical studies have taken a community approach to examine how viral assemblages in host communities vary with land-use change.

In this study, we investigate the effects of deforestation on bat host abundance and diversity, and viral prevalence and richness. We work with bats because they are diverse, abundant, and geographically widespread (Rex et al., 2008), comprising species from nearly every trophic level, with wide differences in their dispersal abilities (Kingston, 2010). Further, some of their life history traits and characteristics (e.g. diet, ability to fly, torpor and hibernations, and roosting behaviors) make them suitable hosts of viruses and other pathogens (Calisher et al., 2006) and many bat species are strongly impacted by land-use changes. Bats are important hosts of pathogens that have had significant impact on public health (e.g. Ebola, SARS, MERS, rabies). They also harbor the highest proportion of zoonotic viruses of any mammal order (Jones et al., 2008; Olival et al., 2017), as well as significant emerging diseases of people, livestock and wildlife. Finally, while no bat viruses have emerged from the Atlantic Forest to our knowledge, we chose this region as our study site because of the high biodiversity it contains and the large-scale deforestation it has undergone. Our study focuses on three questions: (1) Does bat abundance and diversity differ in forested versus non-forested areas? (2) Does viral prevalence differ between bat communities in forested versus deforested areas? (3) What biological and ecological factors determine the likelihood of viral detection?

## Materials and methods

### Ethical statement

This study was carried out with animal handling permits issued from the Brazilian Ministry of the Environment (#33078-4). Animal handling ethics approval was provided by the University of California, Davis (#16048). Bat handling followed strict personal protection and biosafety requirements and short capture times to minimize stress on individual animals. All captured individuals were released at the point of capture.

### Study site

**Figure 1:**
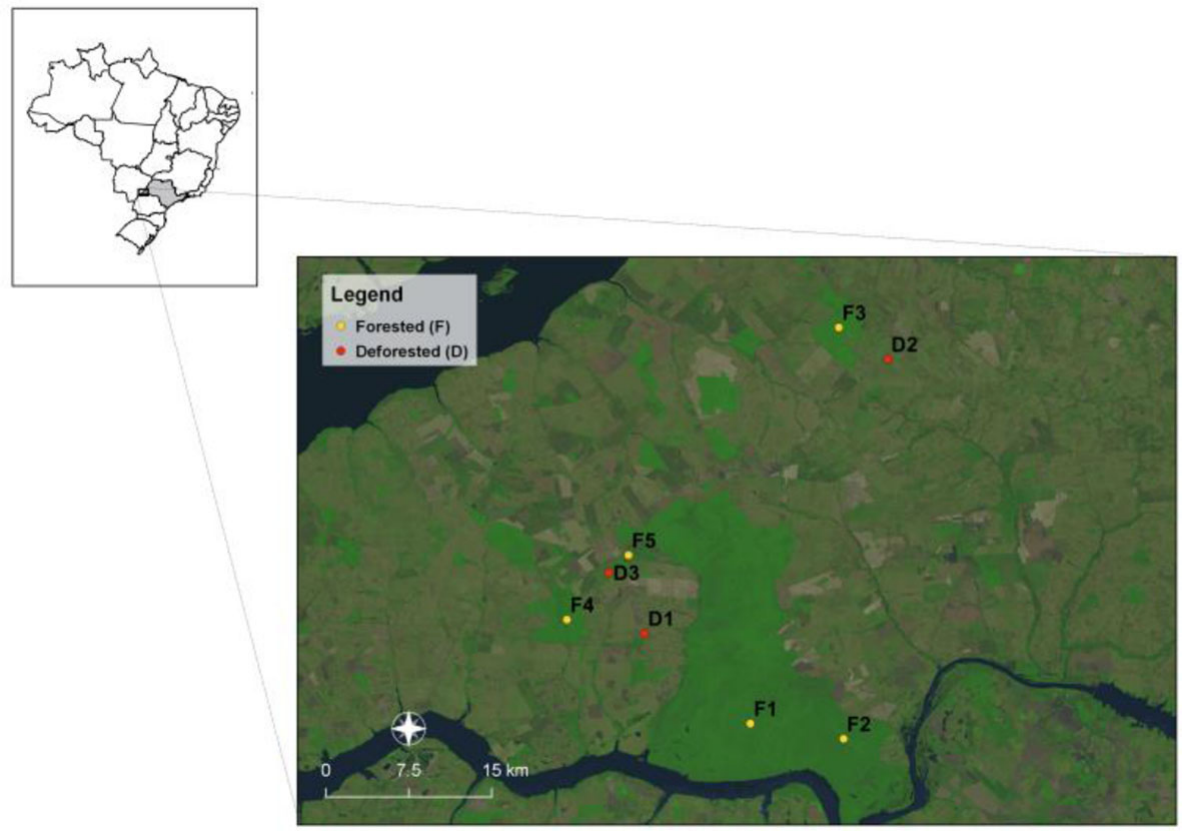
The study area, in and around Morro do Diabo State Park, located in the Pontal do Paranapanema region, Sao Paulo state. Bat surveys (yellow and red circles) were undertaken across forested (n=5) and deforested sites (n=3).

Morro do Diabo State Park (municipality of Teodoro Sampaio, São Paulo state, Brazil, **Figure 1**) is located in the Pontal do Paranapanema region and contains the largest preserved area of interior Atlantic Forest in Sao Paulo State. The park covers an area of 33,845 ha (Durigan and Franco, 2006) and is comprised of mesophytic semideciduous forest and a small area of Cerrado (savanna-like vegetation). The climate is characterized as subtropical, with dry winters and wet summers. Mean annual temperature is 22**°**C, and annual rainfall ranges between 1100 and 1300 mm (de Faria and Pires, 2006). The matrix around the park is comprised of 63 small properties of agrarian reform settlements, as well as pasture (~60%) and agriculture (~15%), and forest fragments ranging from 2 to 2000 ha in area, most of which are privately owned (Uezu and Metzger, 2011). The forested study sites were chosen to control for similar characteristics including elevation, vegetation structure and rainfall. We sampled bats and viruses at five intact forested sites (>200ha) and three nearby deforested sites, located 3-5km away and defined as areas where more than 20% of forest cover has been removed and converted from the original forest to agrarian reform settlements.

### Bat capture and sample collection

Bats were sampled during April to November of 2014. At each site we sampled a 100m x 100m grid using eight horizontal mist nets (9m x 3m), one canopy mist net (6m x 3m) and one harp trap (1.5m x 1.5m). At least 150 individuals were collected from deforested sites and from forested sites each. Additional sampling effort was required in the forested areas due to lower catch rates. Bats were captured for a period of five consecutive nights at each site, totaling 2040 m^2^/270 hours capture effort across all sites. Mist nets were opened at sunset and remained open for six hours. Nets were checked at 30-minute intervals and bats processed immediately.

Samples were collected from bats with no clinical or neurological symptoms and in good body condition, defined as mass divided by forearm length which has been validated in temperate bats (Wilkinson and Brunet-Rossinni, 2009). All animals were released immediately following processing. Blood, saliva, and rectal swabs were collected from each captured animal, with feces and urine opportunistically collected. All samples were placed in cryovials containing 200 ml of Viral Transportation Media (VTM) and stored in liquid nitrogen in a dry shipper while in the field, then transferred to -80C freezers at the Institute of Biomedical Sciences at the University of Sao Paulo. External morphological measurements (including forearm/radius length, body length, head length) were collected by a bat taxonomist to assist in species identification. Several bat identification keys from the region were also used for reference (Reis et al., 2013; Reis et al., 2017). Sex and age were determined by the presence of scrotal testes and well developed teats (Wilkinson and Brunet-Rossinni, 2009). Before release, each individual was marked with a non-toxic pen to determine the rate of within-trip recapture. This was used to ensure that the same bat was not re-sampled within sampling trips.

### Viral detection

Total nucleic acid was extracted from all samples using the EasyMag (bioMérieux, Inc.) platform, and cDNA synthesis performed using SuperScript III first-strand synthesis supermix (Invitrogen), all according to the manufacturer’s instructions. Viral discovery was performed using nested-PCR assays targeting coronaviruses (Quan et al., 2010), astroviruses (Atkins et al., 2009), paramyxoviruses (Tong et al., 2008), and herpesviruses (VanDevanter et al., 1996), while real-time PCR was used to target hantaviruses (Araujo et al., 2011) described below (**Box 1**). PCR results were visualized on a 2% agarose gel and Sanger sequencing was performed using ABI3100 (Applied Biosystems) equipment and BigDye Terminator v3.1 Cycle Sequencing Kit at the Institute of Biomedical Sciences II at the University of Sao Paulo. Sequences were analysed and edited using Geneious (version 6.0.3). Sequences were aligned with ClustalW and MUSCLE, and phylogenetic trees (see Text S1) constructed with neighbor-joining (p-distance, pairwise deletion, 1,000 bootstraps), maximum-likelihood (1,000 bootstraps), and Bayesian (GTR+I - Mr Bayes) algorithms. In Mr. Bayes, we discarded the first 25% of trees as burn-in, and used the remaining trees to estimate the posterior probability value (PP) of 0.7. The chains ran for 2,000,000 cycles (mcmc ngen = 2,000,000). Trees were reconstructed with unconstrained branch lengths and unrooted. In MEGA 7 (macOS available in: https://www.megasoftware.net/) we used Maximum Likelihood with heuristic search and GTR+gamma+I algorithm. For the ML tree, we conducted 1,000 fast bootstrap ML replicates to assess the support values of internal nodes and visualized the trees in FigTree software version 1.4.4 with Midpoint Root (available in: http://tree.bio.ed.ac.uk/software/figtree/)(**Supplementary Figures 1**–**5**). Sequences were segregated into discrete viruses, defined as a viral species, based on distinct monophyletic clustering following Anthony et al. (2013).

**BOX 1. Primers used for viral screening in this study.**

**Table:** 

	**Viral Family**	**Target**	**Amplicon size**	**Primer name**	**Primer sequence 5’- 3’**	**Reference**
Conventional PCR/Semi-Nested/Nested-PCR reaction	**Astroviridae**	RNA-Dependent RNA Polymerase (RdRp)	Round 1 431bp	Astr4380F	GAYTGGRCNCGNTWYGATGGNACIAT	Atkins et al., 2009
				Astr4811R	GGYTTNACCCACATNCCAAA
			Round 2 342bp	Astr4380F + Astr4722R	ARNCKRTCATCNCCATA
	**Coronaviridae**	RNA-Dependent RNA Polymerase (RdRp)	Round 1 520bp	CoV-FWD1	CGTTGGIACWAAYBTVCCWYTICARBTRGG	Quan et al., 2010
				CoV-RVS1	GGTCATKATAGCRTCAVMASWWGCNACATG
			Round 2 328pb	CoV-FWD2	GGCWCCWCCHGGNGARCAATT
				CoV-RVS2	GGWAWCCCCAYTGYTGWAYRTC
	**Herpesviridae**	Polymerase (Pol)	Round 1 variable	DFA	gAY TTY gCN AgY YTN TAY CC
				ILK	TCC Tgg ACA AgC AgC ARN YSg CNM TNA A	Van Devanter et al., 1996
				KG1	gTC TTg CTC ACC AgN TCN ACN CCY TT
			Round 2215-315	TGV	TgT AAC TCg gTg TAY ggN TTY ACN ggN gT
				IYG	CAC AgA gTC CgT RTC NCC RTA DAT
	**Paramyxoviridae**	Polymerase (Pol)	Round 1 639bp	PAR-F1	gAA ggI TAT TgT CAI AAR NTN Tgg AC	Tong et al., 2008
				PAR-R	gCT gAA gTT ACI ggI TCI CCD ATR TTN C
			Round 2 561bp	PAR-R +PAR-F2	gTT gCT TCA ATg gTT CAR ggN gAY AA
SYBRGreen	**Hantaviridae**	S segment	141bp	JAN-F	CCC TgT Tgg ATC AAC Tgg TTT Tg	Araujo et al., 2011
				JAN-R	TgT AAT gTg CTC TTg TTA ACg TCA TCT

### Data analysis

Statistical analysis was performed using R 3.5.1, with ggplot2 for graphing. To compare estimated bat species diversity between forested and deforested sites, we calculated abundance-based diversity profiles with Hill numbers (effective number of species) using the iNEXT package based on the parameter q (Chao and Jost, 2015). This parameter controls the relative emphasis placed on rare or common species. In addition to providing information on species richness, this diversity profile estimator also accounts for species abundances to differing degrees. With increasing order q, the weight of dominant species increases in the calculation of species diversity. We used three widely used species diversity measures: Species richness (number of observed species; q=0), Shannon diversity (number of typical species; q=1) and Simpson diversity (number of most common species; q=2). We then applied a bootstrap method (1,000 bootstraps) using observed detections to obtain approximate variances of the proposed profiles and to construct the associated confidence intervals. These estimations take into account the effect of undetected species in samples. Estimated viral diversity could not be explored using these methods due to limited sample sizes. However we compared viral species richness and overall viral prevalence across treatments using a Fisher’s Exact Test. To account for the uneven number of captures per bat species, we used Bartels rank test of randomness to determine whether viruses were randomly distributed among bat host species by examining whether viral prevalence significantly differed among species. Due to low detection rates in other viral families, our analysis was limited to the *coronaviridae* family.

We use a Generalized Linear model (GLM) of viral detection with a logit link function. We use “viral detection” as the response variable in our model based on the presence or absence of a viral detection for each individual bat. After testing for collinearity among the response variables, no variables were excluded based on their variance inflation factor (VIF) scores. Seven variables were selected for the final analysis. Definitions of the variables used are given in **Table 1**. In a “stepwise backwards-selection”, factors were eliminated from the full model in an iterative process based on the Akaike information criterion (AIC) (Akaike, 1973) with the stepAIC function of the MASS package (Venables and Ripley, 2002) in the statistical software R 3.5.1.

**Table 1:** Description of predictor variables used in the generalized linear models.

Predictor variables	Definition
Treatment	Forested versus Deforested
Sex	Male versus Female
Pregnancy status	Yes or No
Age	Three categories including: juveniles, subadults and adults
Genus	12 unique genera
Species	18 unique species
Abundance	Total number of individuals captured per species

## Results

### Bat diversity

**Figure 2:**
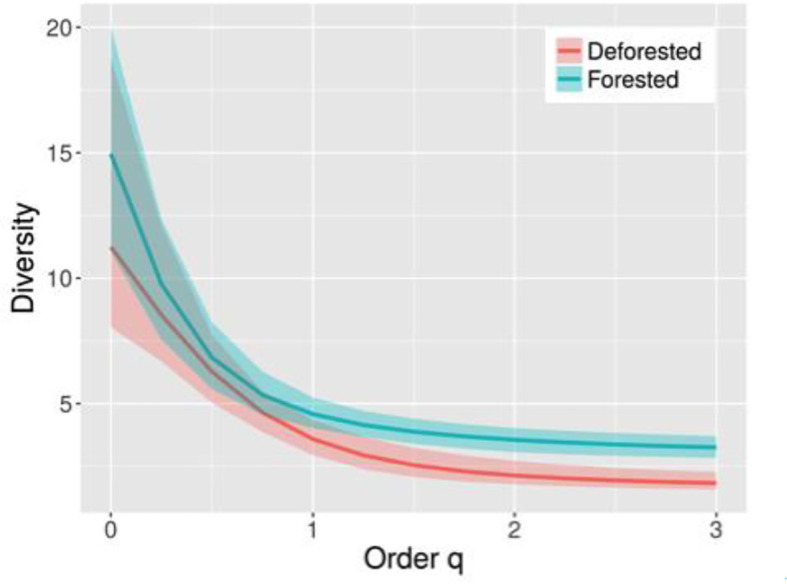
Estimated diversity profiles for bat species data in forested (green line) and deforested (red line) sites for q between 0 and 3 with 95% confidence interval (shaded areas based on a bootstrap method of 1000 replications). The numbers show the estimated diversities for q = 0, 1, 2 and 3.

We recorded 18 bat species from three families (*Phyllostomidae, Molossidae, and Vespertilionidae*) and five dietary guilds (frugivorous, insectivorous, nectarivorous, sanguivorous, omnivourous) from 335 mist-net captures. No bats were captured using the vertical canopy net or harp trap. After accounting for sampling effort, capture rates were similar between forested (n = 163 captures) and deforested (n = 172 captures) sites (Paired t-test, t = 1.883, p = 0.081). Bat species richness in the deforested sites (n=11 species) was slightly higher than the forested sites (n = 9 species); however, this difference was not significant as indicated by the empirical diversity profiles that show overlap between the 95% confidence intervals at q = 0 (**Figure 2**). In contrast, at q>1 (i.e. measures of diversity that incorporate abundance information) the forested sites were found to be more diverse than deforested sites. When correcting for the bias introduced by the non-detection of species in the samples, bat diversity was reduced in deforested sites; species richness was slightly higher in forested areas (n=15 species) compared to deforested areas (n = 11 species). However, for q > 1.25, this difference in community diversity was statistically significant, as reflected by the two non-overlapped confidence intervals.

### Viral prevalence and richness

**Figure 3:**
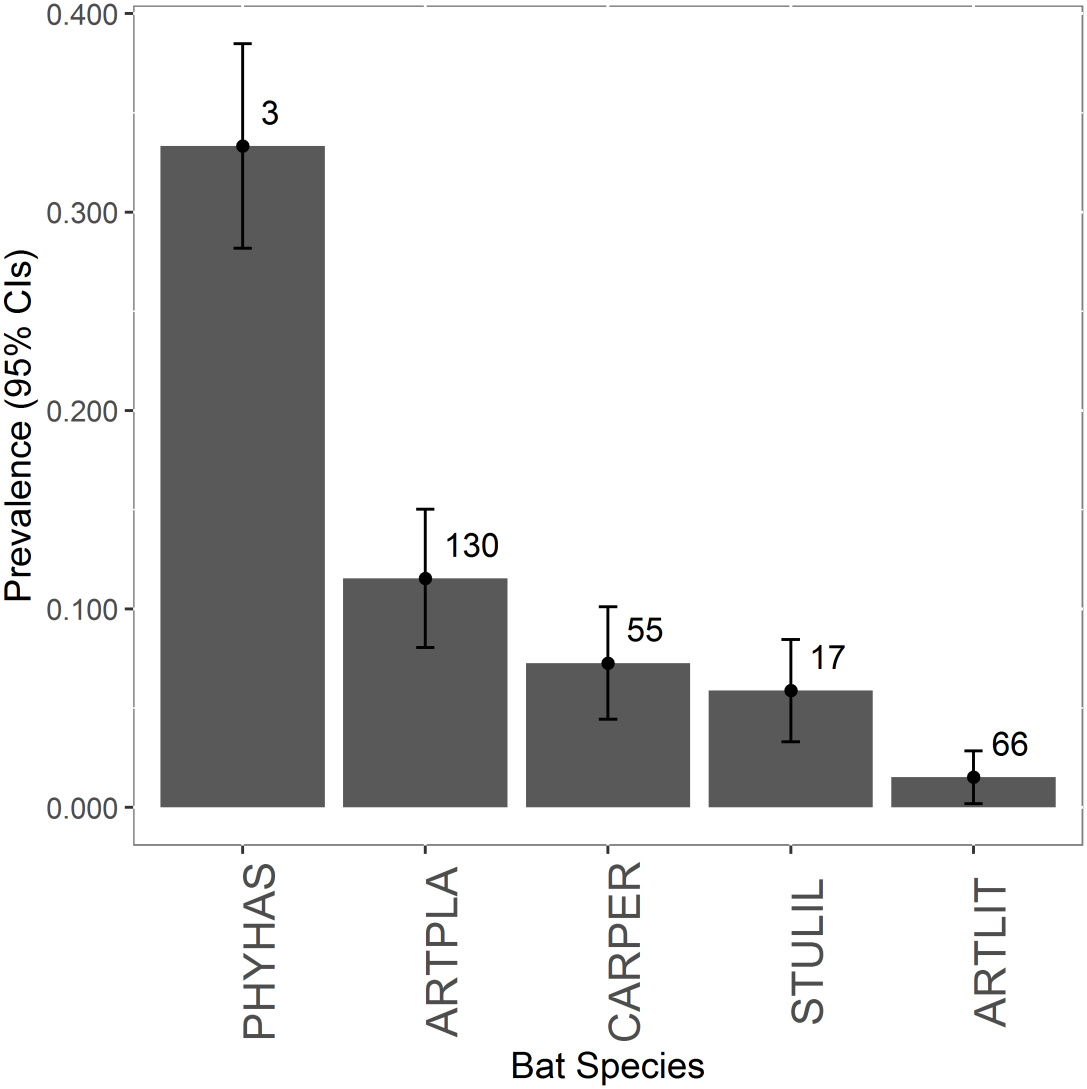
Overall viral prevalence by bat species with sample sizes. Error bars represent the 95% Clopper-Pearson binomial confidence intervals. Species codes: ARTLIT, *Artibeus lituratus*; ARTPLA, *Artibeus planirostris*; CARPER, *Carollia perspicillata*; PHYHAS, *Phyllostomus hastatus*; STULIL, *Sturnira lillium*.

Overall, a total of 22 individual bats from three families (*Phyllostomidae, Molossidae, Vespertilionidae*) were positive for 13 viral species in the following viral families: *Astroviridae*, *Coronaviridae*, *Hantaviridae*, *Herpesviridae*, and *Paramyxoviridae*, with a combined viral prevalence of 6.6% (22/335) (**Table 2**). None of the samples were positive for adenoviruses, despite previous studies documenting their presence in other bat species (Jánoska et al., 2011; Van Vuren et al., 2018). Only one individual bat yielded more than one viral species - a coinfection by a coronavirus and herpesvirus was found in *Artibeus planirostris*. Viral species were not evenly distributed among bat species, with all detected viruses coming from just five of 18 sampled bat species (**Figure 3**). After accounting for the number of captures per bat species by looking at viral prevalence as opposed to number of positive detections, we found a significant effect of host bat species on viral prevalence indicating that viral taxa were detected more frequently in some species than others. In particular, viruses from the *Coronaviridae* family were detected more frequently in generalist species compared to specialist species (P<0.01, Bartel’s Rank Test). Viral prevalence also differed among viral families; *Coronaviridae* had the highest prevalence of 3.6%, followed by *Astroviridae* (1.2%), *Paramyxoviridae* (0.6%), *Herpesviridae* (0.9%) and *Hantaviridae* (0.3%).

**Table 2:** Total captures of bat species and total viral detections in forested and deforested habitat in the Interior Atlantic Forest.

FAMILY/Species	Captures	*Corona-*	*Herpes-*	*Hanta-*	*Astro-*	*Paramyxo-*
PHYLLOSTOMIDAE						
* Artibeus lituratus*	66	1	0	0	0	0
* Artibeus fimbriatus*	34	0	0	0	0	0
* Artibeus planirostris*	130	6	3	1	4	1
* Carollia perspicillata*	55	4	0	0	0	0
* Desmodus rotundus*	1	0	0	0	0	0
* Diaemus youngi*	2	0	0	0	0	0
* Glossophaga soricina*	1	0	0	0	0	0
* Phyllostomus hastatus*	3	0	0	0	0	1
* Sturnira lilium*	17	1	0	0	0	0
* Vampyrodes caracciol*	9	0	0	0	0	0
MOLOSSIDAE						
* Molossus molossus*	3	0	0	0	0	0
* Eumops glaucinus*	2	0	0	0	0	0
VESPERTILIONIDAE						
* Lasiurus blossevillii*	1	0	0	0	0	0
* Myotis nigricans*	1	0	0	0	0	0
* Myotis albescens*	3	0	0	0	0	0
* Myotis riparius*	3	0	0	0	0	0
* Myotis unidentified A*	1	0	0	0	0	0
* Myotis unidentified B*	3	0	0	0	0	0

Treatment (forested vs. deforested), sex and age were the only significant predictors of overall viral detection (presence/absence) (P < 0.05, df = 1) (**Table 3**). This result was supported by the logistic regression model with the lowest AIC value (**Table 4**), which demonstrates that the odds of a positive viral detection decreases in forested habitat.

**Table 3:** Best model of viral detection with different categories of land-use change, sex and age.

	Estimate	SE	z	P value
Intercept	-3.272	0.492	-6.646	3e-11
Treatment (forested vs deforested)	-1.274	0.536	-2.378	0.017
Sex (male vs female)	1.502	0.534	2.812	0.005
Age (juvenile vs adult)	1.247	0.596	2.094	0.036
Age (subadult vs adult)	-0.564	1.067	-0.528	0.597

**Table 4:** Logistic regression model selection table comparing top four models based on lowest AICc.

Model description	AIC	ΔAIC	Null d.f	Residual deviance	Residual d.f.
*~ treatment + sex + age*	6.63	0	333	19.24	329
*~ treatment + sex + age + abundance*	6.92	0.29	333	19.15	328
*~ treatment + sex + pregnancy + age + abundance*	8.29	1.66	333	19.11	327
*~ treatment + species + sex + pregnancy + age + abundance*	31.8	25.1	333	18.52	310

With all viral families combined, viral prevalence in deforested sites (9.3%) was significantly higher than in forested sites (3.68%) (P<0.05, Fisher’s Exact Test). Deforested sites also had higher viral richness (n = 13 unique viral taxa) compared to forested sites (n = 2).

## Discussion

In the Atlantic Forest of Brazil, higher bat host diversity is not associated with higher viral prevalence or richness. Despite lower bat host species richness in deforested areas, viral richness and prevalence is significantly higher. This result does not appear to be associated with the abundance of bat hosts, which was not significantly different (based on mist-net capture frequency) in deforested versus forested areas.

Bats are known to harbor a wide diversity of viruses, and have received growing attention due to their role in the emergence of several recent infectious disease outbreaks (e.g. Severe Acute Respiratory Syndrome (SARS), Middle East Respiratory Syndrome, Nipah virus) (Quan et al., 2010; Hu et al., 2015; Epstein et al., 2020). While studies exploring viral diversity in bat host species have increased, few studies have assessed how bat communities and the viruses they host alter with land-use change, particularly in highly biodiverse sites. Overall, this study identified 13 unique viral taxa from four viral families known to infect humans. We found that different viral families were not evenly distributed within different bat host species and between habitats (forested vs deforested). Specifically, viruses from *Coronoviridae* were primarily found in species considered to be generalists, including *Artibeus planirostris*, *Carollia perspicillata*, *Artibeus literatus*, and *Sturnira lillium*. We suggest that such differences in virus prevalence could be related to viral ecology (i.e., their ability to infect host cells and to persist and replicate) and to the ecology and behavior of the bat hosts in a given habitat. Specifically, we found that viral detection is more likely in juvenile, male bats. Indeed, previous studies have shown that the behavior between many species of young male and female bats differ considerably, with young males immediately leaving the maternity roost once they are weaned, while females continue to forage with their mothers. This difference in behavior could result in younger males having a greater frequency of contacts with new host species or with shared food resources that increase their exposure to potential pathogens. For example, younger vampire bats appear to have higher exposure to pathogens such as rabies virus because younger male bats are more exploratory and are more likely to feed on novel hosts (Carter et al., 2018).

Previous studies of bats have demonstrated that even moderate forest disturbance can result in an increase of certain generalist species that can successfully adapt to human-modified landscapes (Delaval and Charles-Dominique, 2006; Meyer and Kalko, 2008). The strategies they employ such as greater dispersal ability, and the ability to exploit a variety of resources, allow these species to tolerate a wide range of habitats, leading to higher colonization rates throughout human-modified landscapes. In our study area, *A. planirostris and A. fimbriatus* were the two species most commonly captured in deforested sites. Both species are large-bodied frugivores, which feed heavily on figs in the canopy (Handley et al., 1991). In many tropical landscapes, figs are not regularly available throughout the year, thus *Artibeus* species are more likely to occupy disturbed landscapes which provide a variety of food resources (Gorresen and Willig, 2004). *Artibeus* spp. bats accounted for 73% (n=16/22) of all viruses detected, after accounting for the number of captures per bat species.

Here, we show that deforested sites support higher viral richness despite lower bat species richness. While we did not measure disease risk directly, we hypothesize that humans living close to forest edges disturbed by deforestation may be particularly exposed to zoonotic infections not only because of the higher likelihood for humans to be in contact with disease reservoirs, but also because of the higher viral richness found in deforested areas. Yet, previous studies examining the link between land-use change and disease in have been equivocal (Randolph and Dobson, 2012; Salkeld et al., 2013; Civitello et al., 2015; Rulli et al., 2017). Some studies of single-pathogens (e.g. West Nile virus, Hantavirus, the Lyme disease pathogen *Borellia burgdorferi*) in multi-host systems have found that higher pathogen prevalence is associated with decreased continuous forest area (LoGiudice et al., 2003; Suzán et al., 2008; Kilpatrick, 2011). Results from a recent meta-analysis from studies in Southeast Asia shows that people who live or work on agricultural land are more likely to be infected with zoonotic diseases (Shah et al., 2019), and in West and Central Africa, previous research shows that the index cases of Ebola virus outbreaks (i.e. spillover cases from wildlife reservoirs) occurred mostly in areas of forest fragmentation and deforestation (Rulli et al., 2017). Further, urbanized and agricultural areas that have undergone deforestation have been associated with higher rates of disease transmission of West Nile Virus in the United States, increased risk of malaria in Peru (Vittor et al., 2009), Leishmaniasis in Costa Rica (Wijeyaratne et al., 1994) and hantavirus in Panama (Suzán et al., 2008), in part because of changes in host and vector abundance in human-modified areas.

Our study provides further evidence from a multi-pathogen, multi-host species system that deforestation can increase viral prevalence and richness in bat hosts. However, studies of *Plasmodium* infections in Australia (Laurance et al., 2013), Cameroon (Chasar et al., 2009), and Brazil (Ribeiro et al., 2005) found a positive correlation between continuous forest area and pathogen prevalence. In Sabah, Seltmann et al., 2017 found that reduced body mass in bats in logged forests was associated with chronic stress and impaired health status for some species of bats. Interestingly, this did not translate into an increase in coronavirus and astrovirus detection rates among more disturbed sites (Seltmann et al., 2017), perhaps due to the extent of disturbance. Unlike our system, which is more than 30 years post-fragmentation and fully converted, the Sabah study sites are still ongoing active deforestation and fragmentation, which may result in delays in species’ responses.

Our study examines some of the complexities in the relationship among deforestation, viral prevalence and host and viral community assemblages by addressing how viral richness and prevalence in bat hosts varies with land-use change. Our findings suggest that deforestation can increase the abundance of generalist species that, in our case, host the majority of viruses detected. From a theoretical point of view, the dilution effect hypothesis explores how the decrease of biodiversity may increase the amplification of zoonotic diseases. It suggests that high species diversity in a community can reduce infectious disease risk, provided that hosts differ in competency for transmitting a pathogen (Schmidt and Ostfeld, 2001). While this study does not test the ‘dilution effect’ as laid out for Lyme disease and other single pathogen systems (LoGiudice et al., 2003; Kilpatrick et al., 2005), these findings provide further evidence that anthropogenic land use change can in some cases, lead to increased abundance of reservoirs that harbor a higher diversity and prevalence of potential pathogens. As pressures on the environment continue to grow, further research is needed on viral and host ecology and how they are structured across varying landscapes.

## Data availability statement

The original contributions presented in the study are included in the article/**Supplementary Material**. Further inquiries can be directed to the corresponding author.

## Ethics statement 

This study was carried out with animal handling permits from the Brazilian Ministry of the Environment (#33078-4). Animal handling ethics approval was provided by the University of California, Davis (#16048) and by the Ethics Committee of the Faculty of Veterinary Medicine and Zootechnics at the University of Sao Paulo.

## Author contributions

All authors contributed to the article and approved the submitted version.

## Supplementary material

Supplementary Figure 1 Paramyxovirus Maximum Likelihood Phylogenetic Tree. Genetic analysis of 558 nucleotide partial L gene. Tree reconstructed by MEGA7 with heuristic search, Neighbor-Joining “NJ” algorithm and Model GTR+gamma+I. The principal node values superior to 70% represent 1,000 *bootstrap* replicates.

Supplementary Figure 2 Coronavirus Maximum Likelihood Phylogenetic Tree. Genetic analysis of 394 nucleotide partial RdRp gene. Tree reconstructed by MEGA7 with heuristic search, Neighbor-Joining “NJ” algorithm and Model GTR+gamma+I. The principal node values superior to 70% represent 1,000 *bootstrap* replicates.

Supplementary Figure 3 Astrovirus Maximum Likelihood Phylogenetic Tree. Genetic analysis of 369 nucleotide partial RdRp gene. Tree reconstructed by MEGA7 with heuristic search, Neighbor-Joining “NJ” algorithm and Model GTR+gamma+I. The principal node values superior to 70% represent 1,000 *bootstrap* replicates.

Supplementary Figure 4 Herpesvirus Maximum Likelihood Phylogenetic Tree. Genetic analysis of 189 nucleotide partial Polymerase (pol) gene. Tree reconstructed by MEGA7 with heuristic search, Neighbor-Joining “NJ” algorithm and Model GTR+gamma+I. The principal node values superior to 70% represent 1,000 *bootstrap* replicates.

Supplementary Figure 5 Hantavirus Maximum Likelihood Phylogenetic Tree. Genetic analysis of 89 nucleotide partial S segment. Tree reconstructed by MEGA7 with heuristic search, Neighbor-Joining “NJ” algorithm and Model GTR+gamma+I. The principal node values superior to 70% represent 1,000 *bootstrap* replicates.
