# Supplementary figures and images for: Prevalence of bat viruses associated with land-use change in the Atlantic Forest, Brazil
Source: Front Cell Infect Microbiol. 2022 Dec 9;12:921950. doi: 10.3389/fcimb.2022.921950 (PMC9780684; doi:10.3389/fcimb.2022.921950)

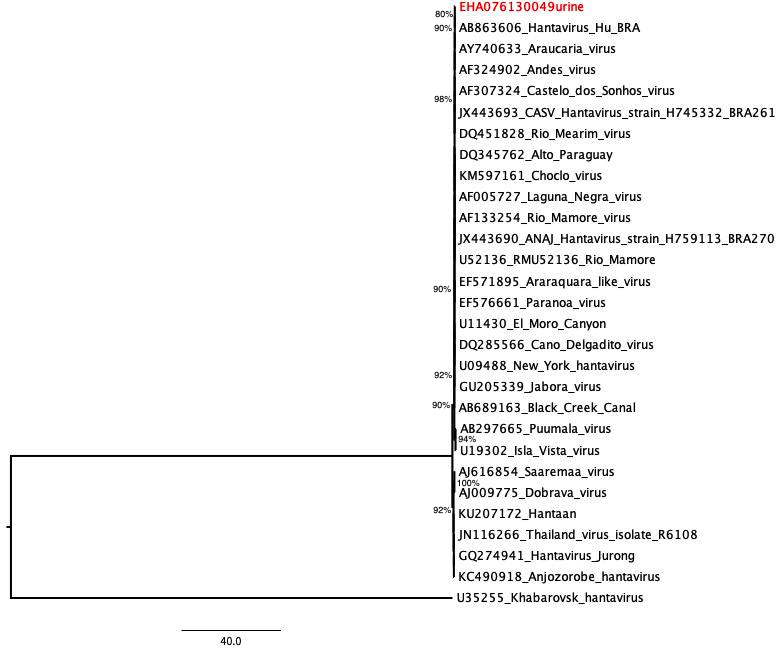

Supplement: Supplementary Figure 1 — Paramyxovirus Maximum Likelihood Phylogenetic Tree. Genetic analysis of 558 nucleotide partial L gene. Tree reconstructed by MEGA7 with heuristic search, Neighbor-Joining “NJ” algorithm and Model GTR+gamma+I. The principal node values superior to 70% represent 1,000 bootstrap replicates. [file Image_1.png]

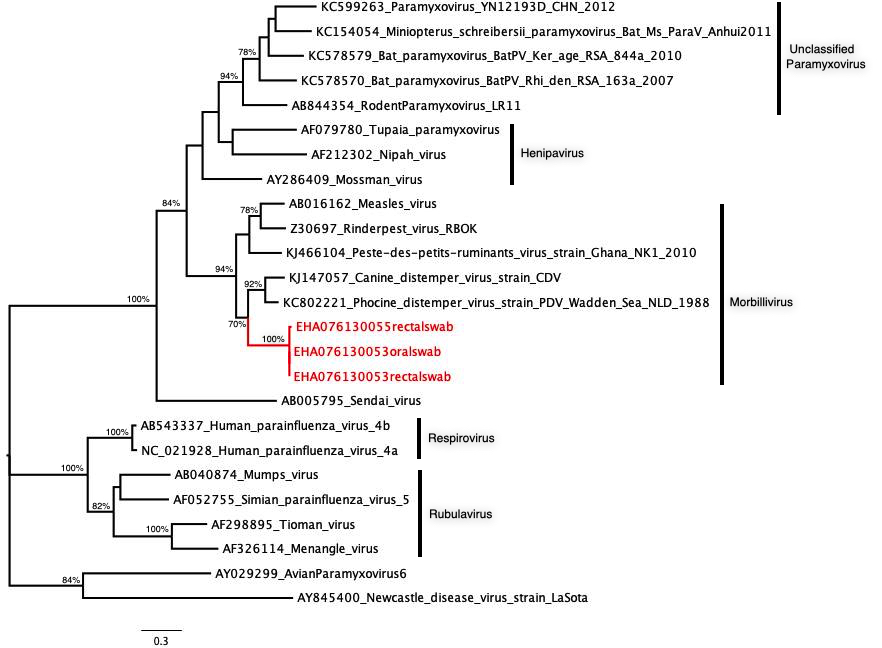

Supplement: Supplementary Figure 2 — Coronavirus Maximum Likelihood Phylogenetic Tree. Genetic analysis of 394 nucleotide partial RdRp gene. Tree reconstructed by MEGA7 with heuristic search, Neighbor-Joining “NJ” algorithm and Model GTR+gamma+I. The principal node values superior to 70% represent 1,000 bootstrap replicates. [file Image_2.jpeg]

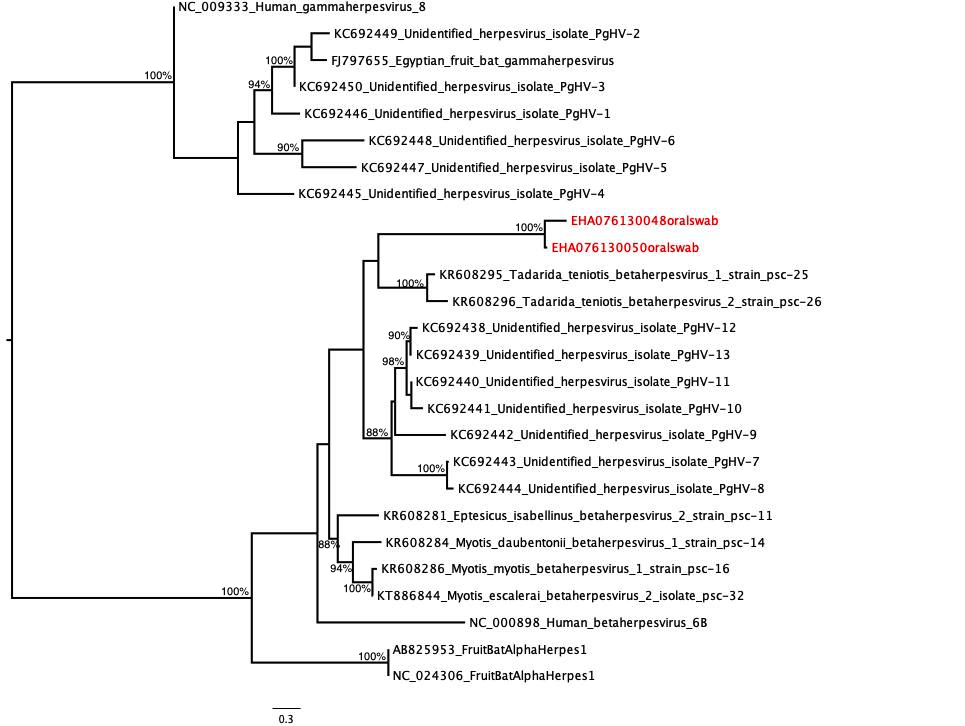

Supplement: Supplementary Figure 3 — Astrovirus Maximum Likelihood Phylogenetic Tree. Genetic analysis of 369 nucleotide partial RdRp gene. Tree reconstructed by MEGA7 with heuristic search, Neighbor-Joining “NJ” algorithm and Model GTR+gamma+I. The principal node values superior to 70% represent 1,000 bootstrap replicates. [file Image_3.png]

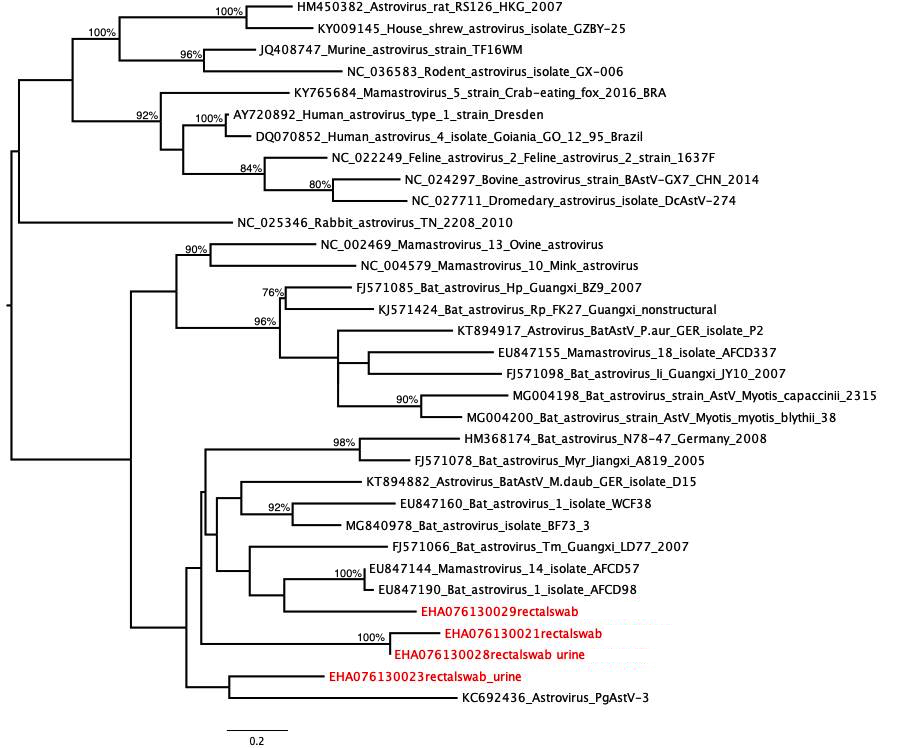

Supplement: Supplementary Figure 4 — Herpesvirus Maximum Likelihood Phylogenetic Tree. Genetic analysis of 189 nucleotide partial Polymerase (pol) gene. Tree reconstructed by MEGA7 with heuristic search, Neighbor-Joining “NJ” algorithm and Model GTR+gamma+I. The principal node values superior to 70% represent 1,000 bootstrap replicates. [file Image_4.jpeg]

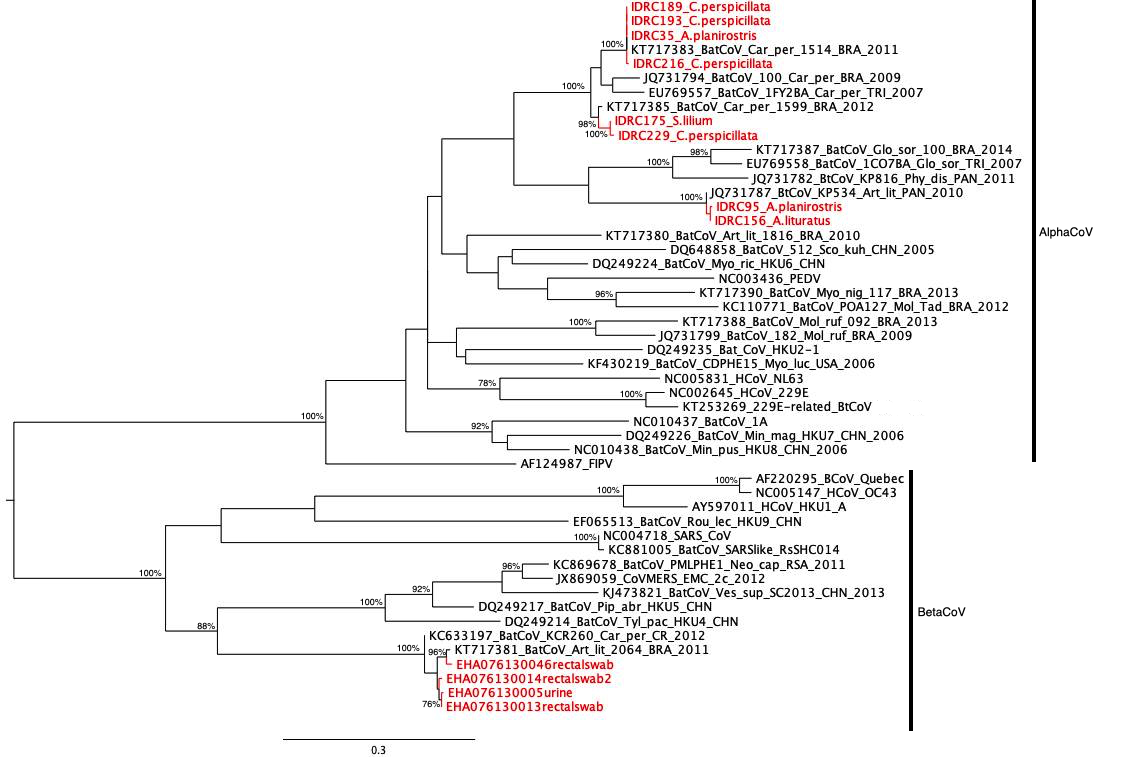

Supplement: Supplementary Figure 5 — Hantavirus Maximum Likelihood Phylogenetic Tree. Genetic analysis of 89 nucleotide partial S segment. Tree reconstructed by MEGA7 with heuristic search, Neighbor-Joining “NJ” algorithm and Model GTR+gamma+I. The principal node values superior to 70% represent 1,000 bootstrap replicates. [file Image_5.png]
